# Comparing different non-invasive methods in assessment of the effects of curcumin on hepatic fibrosis in patients with non-alcoholic fatty liver disease 

**Published:** 2018

**Authors:** Saeede Saadati, Azita Hekmatdoost, Behzad Hatami, Asieh Mansour, Zahra Zahra, Mehdi Hedayati, Amir Sadeghi

**Affiliations:** 1 *Department of Clinical Nutrition and Dietetics, Faculty of Nutrition and Food Technology, National Nutrition and Food Technology, Research Institute, Shahid Beheshti University of Medical Sciences, Tehran, Iran*; 2 *Gastroenterology and Liver Diseases Research Center, Research Institute for Gastroenterology and Liver Diseases, Shahid Beheshti University of Medical Sciences, Tehran, Iran*; 3 *Cellular and Molecular Endocrine Research Center, Research Institute for Endocrine Sciences, Shahid Beheshti University of Medical Sciences, Tehran, Iran *

**Keywords:** Curcumin, Hepatic Fibrosis, NAFLD.

## Abstract

**Aim:**

The aim of this study was to examine the effects of curcumin supplementation on hepatic fibrosis using different fibrosis assessment methods.

**Background:**

Nonalcoholic fatty liver disease (NAFLD) may progress to hepatic fibrosis. Detection of hepatic fibrosis should be measured by liver biopsy, which is an invasive method. Thus, some non-invasive methods are suggested.

**Methods:**

Hepatic fibrosis was evaluated in forty six patients with NAFLD before and three months after supplementation with 1.5 gram curcumin or placebo. Methods of assessments included fibroscan, and calculating non-invasive marker panel including FIB-4 (Fibrosis4), NFS (NAFLD fibrosis score), APRI (AST (Aspartate aminotransferase) Platelet Ratio Index), and BARD (body mass index, AST/ALT (Alanine aminotransferase ratio, diabetes).

**Results:**

Fibrosis score was reduced significantly after curcumin supplementation using fibroscan (p<0.01), FIB-4 (p<0.05) and APRI (p<0.05) tests, while fibrosis score did not change significantly using BARD and NFS methods (p>0.05).

**Conclusion:**

Our results revealed that fibroscan, FIB-4, and APRI are similar in assessment of hepatic fibrosis changes after curcumin supplementation. Future studies with higher sample sizes are needed to confirm these results.

## Introduction

 Nonalcoholic fatty liver disease (NAFLD) encompasses wide spectrum of disorders, from simple steatosis to more aggressive state, steatohepatitis, which can progress to life-threatening states including cirrhosis and hepatocellular carcinoma (1). In light of involvement of the virtue of large number of factors, currently, a multiple-hit hypothesis for rationalizing the pathophysiology of NAFLD is suggested (2). Genetic predisposition along with epigenetic factors, insulin resistance, central obesity, factors related to nutritional and environmental states, as well as gut microbiota are related to its pathogenesis (2). 

Curcumin, a phytoextract from turmeric (*curcuma longa*) (3), with its strong biological and medicinal properties, such as antioxidants, anti-inflammatory and anti-tumor has been more clinically favored (4). In the late 20^th^ century, curcumin was recognized as an active agent for most of the biological activity of turmeric (5). A large body of preclinical literature has uncovered its role in the modulation of mechanisms involved in liver damage (6).

The main goal in achieving the therapeutic treatment of anti- fibrotic agents is that they inhibit the activation of fibrogenic cells, lead to apoptosis of activated hepatic stellate cells (HSC) and prevent extracellular matrix (ECM) proteins deposition (7). Recently, there are no approved agent for treatment and prevention of liver fibrosis, whereas 15-40% of NAFD patients develop hepatic fibrosis (7). With respect to *in-vitr*o and *in-vivo *studies, Curcumin plays a role in inhibiting the activation of HSC by down-regulating of leptin signaling and balance out of formation and degradation of ECM (8).

Non-invasive strategies for assessing fibrosis in patients with NAFLD, is simple and affordable (9). Given the Lancet Commission recommendation (10) of utilizing these risk assessment tools, and lack of human studies in this field, we aimed to assess the effect of Curcumin on hepatic fibrosis through non-invasive marker panel and fibroscan in patients with NAFLD. 

## Methods

**Study Design**

**Figure 1:**
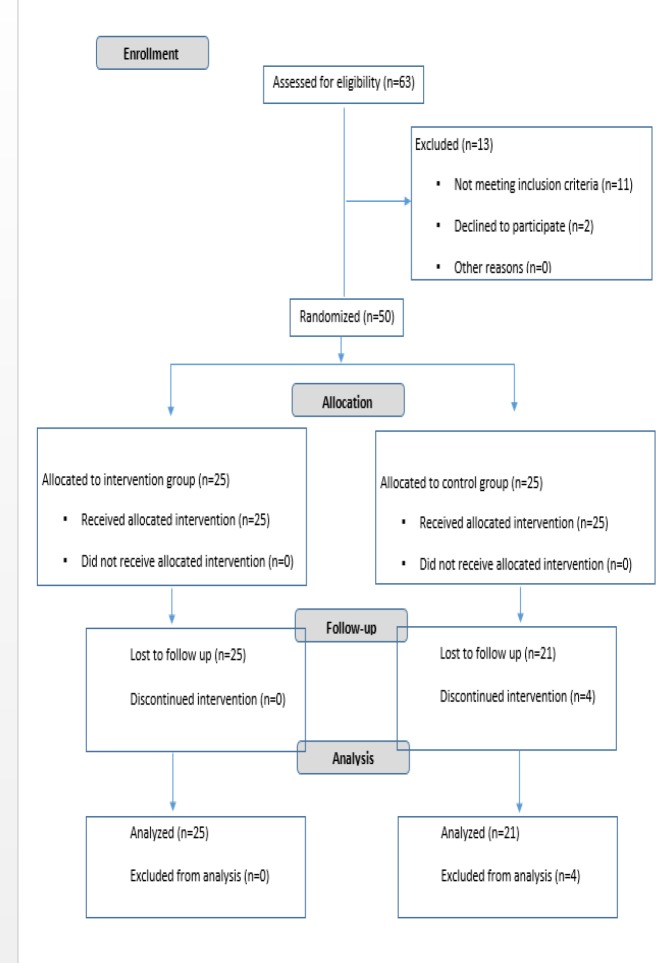
Study design

This randomized controlled clinical trial was conducted from March 2017 to August 2017 (Figure-1). It was approved by the Ethics Committee of National Nutrition and Food Technology Research Institute at Shahid Beheshti University of Medical Sciences and was registered in the Iranian Registry of Clinical Trial (IRCT code: IR.SBMU.nnftri.Rec.1395.106).

According to the results of other studies, fifty patients with NAFLD who had referred to gastroenterology clinics affiliated to Shahid Beheshti University of Medical Sciences were recruited into the study (11,12). It should be noted that 4 participants in the control group were excluded from the study due to lack of desire to continue and undergo surgery operation.

NAFLD was confirmed by an expert physician using fibroscan and laboratory tests. The inclusion criteria of the study were aged 18 years or older with evidence of NAFLD in fibroscan (CAP>263), who did not have the history of alcohol consumption; history of diseases such as liver diseases like hepatitis and cirrhosis, biliary disorders, malignancies; absence of medication consumption such as metformin, vitamin E, ursodeoxycholic acid, phenytoin, tamoxifen, lithium, corticosteroids and methotrexate within previous three months; no history of weight loss or bariatric surgery in recent years; not having history of hypothyroidism and diabetes. The exclusion criteria of this study were pregnancy, supplement intolerance and unexpected adverse side effects.

After obtaining written informed consents, simple randomization according to the table of random numbers was used to allocate patients into intervention (n=25) or control (n=21) groups. Patients in the curcumin group received 3 capsules of 500mg per day, while patients in the placebo group received 3 capsules of placebo, which were obviously similar to the curcumin capsules. Both curcumin and placebo (maltodextrin) capsules produced by Arjuna National Extract, India.

 At first, the researcher explained about the nature of the disease and contributing factors, progression and prognosis, complications and importance of treatment and follow-up. Height and weight of subjects were measured with the accuracy of 0.5 cm and 100 gr, respectively. Measurements were performed in the standing position, with minimal clothing and no shoes at baseline and at 12th week. Waist circumference was measured with tape measure with a precision of 0.5 cm in the state of the narrowest part between the last rib and the iliac bone. Body mass index (BMI) was calculated as weight (kg) divided by height (m) squared.

**Technical Information**

 At the beginning and the end of the study, hepatic fibrosis was assessed using Fibroscan and calculating non-invasive marker panel including FIB-4 (Fibrosis4), NFS (NAFLD fibrosis score), APRI (AST (Aspartate Aminotransferase) Platelet Ratio Index), BARD (body mass index, AST/ALT (alanine aminotransferase ratio, diabetes).

**Statistical Analysis**

Data were expressed as mean ± SD or number (%). Within-group comparisons were performed using paired samples t-test and between-group comparisons were performed using independent samples t-test. Categorical variables were compared using Fisher’s exact test. In order to compare the mean of confounding quantitative variables of anthropometric and dietary measurements in each group, ANCOVA test was used and t-test was used to compare the mean of these two groups. A p-value of <0.05 was considered as statistically significant in all analyses. 

## Results

Fourty-six subjects (25 Curcumin, 21 placebo) completed the study. There were 4 drops out in the control group that all of them were due to the lack of desire to continue owing to the self-perception of that is no longer useful. Dropout rate did not differ between the study groups (p= 0.110). Baseline characteristics of study participants has been shown in Table 1. 

As it is shown in Table 2, body mass index (BMI), waist to hip ration (WHR), energy intake and rate of physical activity considered as metabolic confounders based on literature reviews. Energy intake and consequently BMI in both groups significantly decreased during the study, although there was no significant difference between the two groups. In addition, physical activity increased in both groups, since all the participants were advised to increase physical activity; however, this increase was not statistically significant. The WHR was significantly reduced only in the group receiving curcumin (Table2).

As it is shown in table 3, Curcumin reduced fibrosis score in fibroscan (p<0.01), FIB-4 (p<0.05) and APRI (p<0.05) tests, while fibrosis score did not change significantly using BARD and NFS(p>0.05). Also, comparison of these indices between the two groups showed a significant difference in FIB-4.

In order to eliminate the effects of confounding factors, either in the beginning or during the study, the analysis of covariance test was used. All ANCOVA models were adjusted for the baseline value of each variable and mean changes in BMI, WHR, MET and energy. At the end of the study, no significant improvement in fibrosis indices was observed between groups, after the adjustment for BMI, MET, WHR and energy intake (Table 4).

**Table 1:** Baseline characteristics at enrollment^1^

Characteristics	Curcumin group (n = 25)	Control group (n = 21)	a *P*-value
Age (y)	46.64 ± 11.7 2	45.33 ± 11.47	0.705
Sex (M/F)	11/14	12/9	0.277
Height (cm)	161.8 ± 8.75	166.1 ± 11.9	0.179
Weight (kg)	84.96 ± 11.56	88.34 ± 13.31	0.362
WC (cm)	102.32 ± 9.08	101.43 ± 8.56	0.735
HC (cm)	113.92 ± 9.37	111.33 ± 7.66	0.317
Fibrosis score (KPa)	6.51 ± 1.91	6.61 ± 2.43	0.277
FIB4	1.15 ± 0.67	0.85 ± 0.31	0.163
NFS	1.58 ± 0.78	1.15 ± 0.40	0.286
APRI	0.27 ± 0.15	0.24 ± 0.08	0.474
BARD	1.76 ± 0.91	1.73 ± 0.96	0.591

1 WC, waist circumference; HC, hip circumference; FIB-4, Fibrosis4; NFS, NAFLD Fibrosis Score; APRI, AST(Aspartate Aminotransferase) Platelet Ratio Index; BARD,body mass index, AST/ALT ratio,diabetes;

2 Mean ± SD (all such values);

a Student’s t-test

**Table 2:** Comparison of the two groups’ mean ± SD of metabolic confounders before and after the intervention^1^

	Curcumin group (n = 25)		Control group (n = 21)		a *P*-value
	Wk0	Wk12	Wk0	Wk12	
BMI (kg/m2)	32.57 ± 4.64	31.78 ± 5.07***	32.19 ± 5.22	31.18 ± 6.38***	0.261
WHR	0.89 ± 0.06	0.87 ± 0.06**	0.91 ± 0.06	0.91 ± 0.05*	0.350
Energy intake (Kcal/day)	2369 ± 702	1843 ± 467***	2284 ± 535	1903 ± 573**	0.831
MET.h/d	33.67 ± 4.79	34.13 ± 4.33*	32.84 ± 5.81	34.43 ± 4.56*	0.789

1 BMI, body mass index; WHR, waist to hip ratio; MET, metabolic equivalent of tasks.

2 Mean ± SD (all such values);

a Student’s t-test for differences; Paired t-test:

* NS ,

** p-value <0.05 ,

*** p-value<0.01

**Table 3:** Comparison of the two groups’ mean ± SD of liver fibrosis scores before and after the intervention^1^

	Curcumin group (n = 25)		Control group (n = 21)		a *P*-value
	Wk0	Wk12	Wk0	Wk12	
Fibrosis score	6.51 ± 1.91	5.61 ± 1.42***	6.61 ± 2.43	6.06 ± 1.85*	0.320
FIB4	1.15 ± 0.67	0.70 ± 0.30**	0.85 ± 0.31	0.80 ± 0.24*	0.042
NFS	1.58 ± 0.78	1.39 ± 0.82*	1.58 ± 0.78		0.586
APRI	0.24 ± 0.08	0.19 ± 0.05*	0.27 ± 0.15		0.678
BARD	1.73 ± 0.96	1.13 ± 0.91*	1.76 ± 0.91		0.488

1 FIB-4 (Fibrosis4); NFS (NAFLD fibrosis score), APRI (AST (Aspartate Aminotransferase) Platelet Ratio Index); BARD (body mass index, AST/ALT ratio, diabetes);

2 Mean ± SD (all such values);

a Student’s t-test for differences;Paired t-test:

* NS ,

** p-value <0.05 ,

*** p-value<0.01

**Table 4:** Mean changes (95% CI) from baseline in liver fibrosis scores by treatment group 1

Change from baseline	Curcumin group (n = 25)	Control group (n = 21)	P value 2
Fibrosis score (KPa)	-0.89 (-1.30, -0.49)	-0.55 (-1.17, 0.06)	0.296
FIB4	-0.45 (-0.82, -0.07)	-0.04 (-0.17, 0.09)	0.428
NFS	-0.18 (-0.5, 0.13)	-0.07 (-0.32, 0.19)	0.661
APRI	-0.05 (-0.09, -0.005)	-0.04 (-0.08, 0.003)	0.786
BARD	-0.32 (-0.82, 0.18)	-0.60 (-1.29, 0.08)	0.282

1 FIB-4, Fibrosis4; NFS, NAFLD Fibrosis Score; APRI, AST(Aspartate Aminotransferase) Platelet Ratio Index; BARD,body mass index, AST/ALTratio,diabetes;

2 Based on an ANCOVA model that regressed changes from baseline on treatment group, baseline value of the outcome, and mean changes in BMI, WHR, MET and energy.

## Discussion

The present study suggested that a significant benefit of Curcumin supplementation in improving hepatic fibrosis examined by fibroscan, Fib-4, and APRI, but not in BARD, and NFS. To the best of our knowledge, this is the first human study compared different non-invasive methods in assessment of the effects of Curcumin on hepatic fibrosis in Patients with NAFLD. It is known that the main mechanism underlying hepatic fibrosis is through activation of HSCs. *In-vivo *studies have shown that Curcumin significantly reduced size of adipocytes, number of macrophages and mast cells and alter collagen position in adipose tissue. These studies have reported that Curcumin acts as an anti-fibrotic agent to prevent the progression of liver disease in obese mice (13). These studies results are in line with our results using fibroscan, Fib-4, and APRI scoring exams.

We used different suggested scoring exams to evaluate hepatic fibrosis including non-invasive marker panel such as NFS (14), FIB-4 (15), BARD (16), and APRI (17), as well as, fibroscan (18-20). Although liver biopsy is the gold standard for evaluation of hepatic fibrosis, it cannot be used as a routine screening tool (21) because of its invasive nature and probable side effects. Thus, these non-invasive methods have been suggested for not only evaluation of disease progression, but also for monitoring response rate to anti fibrotic agents (22). There are several studies that have shown the reliability and validity of fibroscan test for assessment of hepatic fibrosis; however, its cost is not affordable for many of the patients. Thus, we compared the results of other non-invasive methods to fibroscan, as a known valid non-invasive method. Our results showed the same results using fibroscan, Fib-4, and APRI, while the results were different using NFS and BARD methods.

Considering fibroscan as a standard method of fibrosis assessment, our results confirmed that FIB-4 and APRI can be used for fibrosis assessment when fibroscan is not affordable in the clinical setting.

 The main limitation of the present study was that we could not perform liver biopsy as the gold standard to compare other noninvasive screening tools with it owing to ethical issues. Besides, given the small size of participants, there is a need for further studies with larger sample size. 

In conclusion, our results revealed that FIB-4 and APRI can be used for fibrosis assessment when fibroscan is not affordable in the clinical setting. Further studies are warranted to elucidate these effects in larger population.
